# Development and validation of a prognostic nomogram for unresectable pancreatic ductal adenocarcinoma with synchronous liver metastases: a study based on the SEER database and an external cohort

**DOI:** 10.3389/fonc.2025.1636715

**Published:** 2025-08-27

**Authors:** Lu Huan, Qi He, Yang Cao, Changan Liu, Yue Li

**Affiliations:** ^1^ Department of Hepatobiliary Surgery, The Second Affiliated Hospital of Chongqing Medical University, Chongqing, Chongqing, China; ^2^ Department of Hepatobiliary and Pancreatic Surgery, Renji Hospital, School of Medicine, Chongqing University, Chongqing, China

**Keywords:** pancreatic ductal adenocarcinoma, synchronous liver metastases, unresectable cancer, nomogram, prognostic factors

## Abstract

**Background:**

Pancreatic ductal adenocarcinoma with synchronous liver metastases (PDACLM) represents a highly aggressive malignancy with limited treatment options and poor prognosis. While conversion therapy may enable curative surgery in a small subset of patients, the majority ultimately remain ineligible for resection. Prognostic tools tailored to this unresected population are lacking but urgently needed for guiding clinical decisions.

**Methods:**

We conducted a retrospective cohort study using 9,469 patients with histologically confirmed PDACLM from the SEER database (2010–2015), supplemented by an external validation cohort of 94 patients treated at the Second Affiliated Hospital of Chongqing Medical University (2020–2023). Multivariate Cox regression analysis was used to identify independent prognostic factors. A nomogram was constructed and validated internally and externally to predict individualized overall survival (OS) at 12, 18, and 24 months.

**Results:**

Age ≥65 years, higher tumor grade, and undetermined nodal status were independently associated with reduced OS, while chemotherapy, radiotherapy, and metastasis-directed surgery significantly improved survival outcomes (all P<0.05). The nomogram demonstrated good discriminative performance with a C-index of 0.73 in the training cohort and 0.72 in internal validation. External validation showed consistent predictive accuracy (C-index: 0.715). Calibration plots and decision curve analyses supported the model’s reliability and clinical utility.

**Conclusion:**

We developed and externally validated a clinically accessible nomogram for survival prediction in unresected PDACLM patients. This tool may assist clinicians in risk stratification and treatment planning for a frequently overlooked patient subgroup. Further prospective validation is warranted to confirm its applicability in broader clinical settings.

## 1 Introduction

Pancreatic ductal adenocarcinoma (PDAC) is a highly lethal malignancy with poor prognosis (1, 2). As of Cancer Statistics 2024, it ranks fourth in cancer-related mortality, with a 5-year survival rate below 10% and limited advances for metastatic cases (1). Surgery is the only curative approach, yet only ~20% are eligible; among them, over 80% face recurrence or metastasis within two years, despite a 5-year survival of about 21% (3–5). Over half of PDAC patients develop distant metastases, which significantly worsens outcomes (6, 7). At diagnosis, 50–60% present with synchronous metastases, mainly to the liver, peritoneum, and lungs, leading to rapid decline (8, 9). Even with regimens like FOLFIRINOX or gemcitabine/nab-paclitaxel, those with liver metastases typically survive only 3 to 6 months (10, 11).

In a cohort of 13,233 PDAC patients with distant metastases, Oweira et al. reported that 76% had liver involvement (12). Surgical resection, radiotherapy, and radiofrequency ablation have shown potential benefits for selected patients with PDAC liver metastases (PDACLM) (13–15). However, many ultimately become ineligible for surgery due to complex clinical factors. While the American Joint Committee on Cancer (AJCC) TNM system is used to stage PDAC, it lacks a tailored model for predicting outcomes and risk in PDACLM (16). Conversion therapy has recently gained attention as it allows a subset to undergo surgery after good response (17), also NAT increases resectability and R0 resection rates (18). These patients are often excluded from current prognostic models focused on surgical outcomes (19). Moreover, existing tools frequently depend on molecular or imaging biomarkers that are not widely accessible or validated across populations (20). This results in a significant gap in individualized risk evaluation for advanced, unresectable PDAC patients, making survival stratification essential for treatment planning and patient communication.

Most landmark studies and existing prognostic nomograms focus on patients who achieve surgical resection after conversion therapy, thereby excluding those who remain unresectable and reinforcing a “resectability bias” that obscures key survival determinants in advanced disease (21, 22), To address this gap, we performed a large-scale retrospective study using data from the U.S.-based SEER database and an independent external cohort to identify prognostic factors in PDACLM patients who did not receive curative surgery. Using these variables, we developed and validated a clinically applicable nomogram to predict personalized survival outcomes. This tool is intended to support multidisciplinary decision-making and improve prognostic assessment in this often-overlooked patient group.

## 2 Methods

The authors obtained authorized access to the SEER database (Incidence – SEER Research Data, 17 Registries, November 2024 Submission) via SEER*Stat software (Account No. 22821-Nov2020). All data were downloaded prior to April 4, 2025, in accordance with the data use agreement and policies issued by the U.S. National Cancer Institute (NCI). No restricted or custom-linked datasets were used. Informed consent was not required for SEER data, as these data are publicly accessible at: https://seer.cancer.gov/data/.

This study was conducted in full compliance with the ethical standards of the 1964 Helsinki Declaration and its later amendments. The external cohort study was reviewed and approved by the Ethics Committee of The Second Affiliated Hospital of Chongqing Medical University(Approval No. 2025-YLS-023). As this was a retrospective analysis of anonymized records, the requirement for written informed consent was formally waived.

### 2.1 Data source and patient selection

Clinicopathological and survival data of patients diagnosed with PDACLM between 2010 and 2015 were extracted from the Surveillance, Epidemiology, and End Results (SEER) database using SEER*Stat software. Patients were identified using the International Classification of Diseases for Oncology, Third Edition (ICD-O-3) site codes C25.0–C25.3 and C25.7–C25.9, and limited to those with histologically confirmed adenocarcinoma (ICD-O-3 histology codes 8140/3 and 8500/3) to ensure diagnostic consistency (23, 24). Only patients with liver metastases present at initial diagnosis were included, while those with metastases to other distant sites (lung, brain, or bone) were excluded. Since SEER began recording organ-specific metastasis data in 2010, cases prior to this time were not considered.

Additional inclusion criteria were as follows: (1) age ≥18 years at diagnosis; (2) absence of recommendation for curative surgical resection, or inability to undergo such treatment. Exclusion criteria included: (1) history of other primary malignancies; (2) incomplete records regarding survival time, metastatic sites, or clinical staging variables; and (3) overall survival time of less than one month.

To externally validate the robustness and generalizability of the prognostic model, an independent cohort of 94 patients with pathologically confirmed PDACLM diagnosed between January 2020 and December 2023 was retrospectively collected from The Second Affiliated Hospital of Chongqing Medical University. Follow-up information was primarily obtained through outpatient records, electronic health information systems and telephone interviews with patients’ family members, with death confirmed by official death certificates. Among them, 12 deceased patients were excluded due to the lack of accurate survival time or with an extremely short survival time, resulting in 82 patients being included in the final external validation cohort. Overall survival (OS) was defined as the time from diagnosis to death or the last follow-up. Follow-up was completed in January 2024 (**Supplementary Figure 1**).

### 2.2 Variables collected

Data points extracted from the patient cohort included: Patient characteristics (age, sex, vital status, survival time), tumor characteristics (Histological type, laterality, primary site, grade of differentiation, AJCC stage, T stage, N stage), and Additional treatment (radiotherapy and chemotherapy) and surgical information. In the analysis patients of specific age at diagnosis were classified into two groups (<65y and ≥65y) according to accepted cut-off values.

### 2.3 Missing data handling and multiple imputation

A large proportion of patients in the SEER cohort (78.3%) had missing values for tumor grade. To minimize potential bias and loss of statistical power, missing grade values were addressed using multiple imputation by chained equations (MICE). The imputation model incorporated relevant covariates, including age, sex, tumor site, T stage, N stage, overall survival time, and survival status, under the assumption that data were missing at random (MAR). Ten imputed datasets were generated, and results were combined using Rubin’s rules for subsequent Cox regression modeling and nomogram development. Diagnostic evaluations were conducted to ensure the validity and robustness of the imputation. Specifically, the distribution of grade values before and after imputation was compared, pairwise correlations between clinical variables were assessed, and Kaplan–Meier survival curves stratified by tumor grade were plotted pre- and post-imputation.

### 2.4 Construction of the nomogram

Survival times for categorical variables were compared using the log-rank test in univariate analysis, and survival curves were drawn using the Kaplan–Meier method. The variables with *P* values of<0.05 were then subjected to multivariate cox regression analysis to screen for risk factors and independent prognostic factors for overall survival (OS) in the training cohort, and hazard ratios (HR) and corresponding 95% confidence intervals (95% CI) for the variables were calculated. Based on these independent prognostic factors, we used the statistical software to establish a nomogram to predict the probability of OS rates at 1, 1.5 and 2years in PDACLM patients who were ultimately unable to receive curative resection.

### 2.5 Performance metrics and decision curve validation of the nomogram

The effectiveness of the nomogram in distinguishing outcomes was assessed through the concordance index (C-index) and time-dependent receiver operating characteristic (ROC) curves, with the area under the curve (AUC) determined for OS at 12, 18, and 24 months. The C-index varies between 0.5 and 1.0, with increased values signifying enhanced predictive precision; a C-index exceeding 0.7 typically suggests an adequate level of discriminatory capacity (25). Simultaneously, Time-dependent C-index and Brier scores were calculated using the riskRegression and pec packages to evaluate the model’s discriminative and calibration performance over time. These metrics were calculated in the training cohort and validated in both the SEER internal validation set and an external validation cohort.

Calibration of the nomogram was assessed by calibration plots generated using 1,000 bootstrap resamples, comparing predicted survival probabilities with actual outcomes. A perfect prediction would align with a 45-degree diagonal reference line. Separate calibration curves were plotted for 12-, 18-, and 24-month OS in each cohort, and their proximity to the ideal line was used to assess prediction accuracy.

In order to assess the clinical applicability of the nomogram, a decision curve analysis (DCA) was performed. This analytical approach quantifies the net benefit of a predictive model over a spectrum of threshold probabilities, while considering the associated risks of false positives and false negatives. Within this investigation, DCA curves were generated for each of the three cohorts, allowing for a comparative evaluation of the nomogram’s efficacy against the conventional “treat-all” and “treat-none” approaches (26). Additionally, the net benefit of the nomogram was contrasted with the traditional AJCC TNM staging system to assess whether the nomogram offered superior clinical decision-making support.

### 2.6 Statistical analysis

To address potential treatment imbalances, especially the higher rate of radiotherapy in the external validation cohort, we conducted several supplementary analyses. First, we performed propensity score matching (PSM) between the SEER training set and external cohort to control for baseline discrepancies including treatment exposure. Additionally, stratified Kaplan–Meier analyses and a multivariable Cox model with interaction terms (Cohort × Radiotherapy) were used to explore potential effect modification. These analyses aimed to assess whether radiotherapy alone could explain the observed survival patterns and to verify the model’s robustness across subgroups. Furthermore, to address concerns regarding cohort imbalance and potential distributional bias, we conducted sensitivity analyses using subsampling techniques. Specifically, stratified undersampling and oversampling were applied when calculating the ROC curves for the training, internal validation, and external cohorts. This allowed a more balanced evaluation of model performance across varying distributions and case counts. Besides, Ten-fold cross-validation was performed on the SEER training set to assess internal reproducibility and minimize overfitting, with the C-index calculated in each fold to evaluate model robustness.

## 3 Results

### 3.1 Study cohort

9,469 PDACLM ptients from 2010–2015 were extracted from the SEER database; in addition, 82 PDACLM patients from 2020–2023 were included as an external validation cohort from the Second Affiliated Hospital of Chongqing Medical University, China. Patients in the SEER database were randomly divided into the training cohort (*n*=6,628) and the internal validation cohort (*n*=2,841) according to the ratio of 7:3. In the training cohort, 3,685 (55.6%) males and 2,943 (44.4%) females were diagnosed with the age (<65y: 41.58% and ≥65y: 58.42%). Regardless of whether they underwent conversion therapy or not, these patients ultimately did not meet the criteria for curative surgical treatment, or they did not receive the final curative surgical treatment. And of these patients, 5,487 patients received chemotherapy, 318 patients received radiotherapy, and 216 patients underwent treatment for metastatic lesions (such as ablative therapies). **Table 1** shows the demographic and clinicopathological characteristics of the training, internal and external validation groups.

**Table 1:** Demographics and clinicopathologic characteristics of the training and external validation cohort.

Characteristics	Training cohort (N=6628)	Internal validation cohort (N=2841)	External validation cohort (N=82)
Age			
< 65y	2756 (41.58%)	1142 (40.20%)	41 (50.00%)
≥ 65y	3872 (58.42%)	1699 (59.80%)	41 (50.00%)
Sex			
Male	3685 (55.60%)	1566 (55.12%)	55 (67.07%)
Female	2943 (44.40%)	1275 (44.88%)	27 (32.93%)
Site			
Head of pancreas	2714 (40.95%)	1151 (40.51%)	27 (32.93%)
Body or tail of pancreas	2562 (38.65%)	1115 (39.25%)	55 (67.07%)
Others	1352 (20.40%)	575 (20.24%)	0 (0.00%)
Grade			
Grade I	154 (2.32%)	56 (1.97%)	0 (0.00%)
Grade II	515 (7.77%)	228 (8.03%)	13 (15.85%)
Grade III	733 (11.06%)	347 (12.21%)	12 (14.63%)
Grade IV	36 (0.54%)	15 (0.53%)	3 (3.66%)
Unknown	5190 (78.30%)	2195 (77.26%)	54 (65.85%)
T			
T1	251 (3.79%)	94 (3.31%)	1 (1.22%)
T2	2562 (38.65%)	1068 (37.59%)	31 (37.80%)
T3	2308 (34.82%)	1034 (36.40%)	32 (39.02%)
T4	1507 (22.74%)	645 (22.70%)	18 (21.95%)
N			
N0	3719 (56.11%)	1627 (57.27%)	40 (48.78%)
N1	2180 (32.89%)	919 (32.35%)	42 (51.22%)
NX	729 (11.00%)	295 (10.38%)	0 (0.00%)
SurgOthSites			
No	6484 (97.83%)	2769 (97.47%)	57 (69.51%)
Yes	144 (2.17%)	72 (2.53%)	25 (30.49%)
Radiation			
No	6412 (96.74%)	2739 (96.41%)	64 (78.05%)
Yes	216 (3.26%)	102 (3.59%)	18 (21.95%)
Chemotherapy			
No	2772 (41.82%)	1210 (42.59%)	31 (37.80%)
Yes	3856 (58.18%)	1631 (57.41%)	51 (62.20%)

SurgOthSites, Surgery of other sites.

### 3.2 Missing data imputation results

Following multiple imputation of missing tumor grade values, the distribution of grade levels became more consistent with known epidemiological patterns, with Grade 3 remaining the most prevalent subtype. Correlation analyses indicated improved associations between imputed grade and key clinical variables, such as survival status and nodal stage. In addition, Kaplan–Meier survival curves plotted before and after imputation showed preserved and even enhanced separation across grade levels, supporting the clinical validity of the imputed values. These findings confirmed that the imputation procedure strengthened the overall robustness of the prognostic model without distorting the relationship between tumor grade and patient survival (**Supplementary Figure 2**).

### 3.3 Independent prognostic factors

Multivariate Cox regression analysis identified several independent prognostic factors associated with overall survival in patients with PDACLM who did not undergo curative surgery (**Table 2**). Older age (≥65 years) was significantly correlated with worse survival (HR=1.256, 95% CI: 1.195–1.321, *P*<0.001). Tumor grade showed strong prognostic relevance: compared to Grade I, patients with Grade II (HR=2.369), Grade III (HR=3.318), Grade IV (HR=2.480), and unknown grade (HR=2.903) all had significantly increased mortality risk (*all P*<0.001). Lymph node status also played a role, with patients classified as NX showing poorer outcomes than node-negative individuals (HR=1.120, *P*=0.006). Among NX patients, a majority (approximately 70%) were classified as grade III–IV, suggesting that unknown lymph node status may be associated with more advanced tumor characteristics. Treatment-related variables, including surgery at other metastatic sites (HR=0.804, *P*=0.011), radiotherapy (HR=0.858, *P*=0.028), and chemotherapy (HR=0.420, *P*<0.001), were all associated with improved survival. In contrast, sex, tumor location, and T stage were not independently significant. These results highlight that age, tumor differentiation, nodal evaluation, and multimodal treatment approaches—particularly chemotherapy—are critical determinants of survival in this advanced disease setting. The results of the univariate and multifactorial analyses are shown in **Table 2**.

**Table 2:** Selected factors in the training cohort for building the model by univariate and multivariate Cox regression analysis.

Characteristics	Uni-HR	Uni-CI	Uni-P	Multi-HR	Multi-CI	Multi-P
Age						
< 65y	Reference					
≥ 65y	1.382	1.315-1.453	0	1.256	1.195-1.321	0
Sex						
Male	Reference					
Female	1.015	0.967-1.066	0.55			
Site						
Head of pancreas	Reference					
Body or tail of pancreas	1.015	0.961-1.072	0.593			
Others	1.066	0.998-1.138	0.058			
Grade						
Grade I	Reference					
Grade II	2.028	1.676-2.453	0	2.369	1.958-2.867	0
Grade III	2.764	2.298-3.325	0	3.318	2.757-3.994	0
Grade IV	2.111	1.442-3.089	0	2.48	1.694-3.631	0
Unknown	2.453	2.066-2.913	0	2.903	2.444-3.449	0
T						
T1	Reference					
T2	1.099	0.964-1.253	0.16			
T3	1.043	0.914-1.19	0.532			
T4	1.071	0.935-1.226	0.323			
N						
N0	Reference					
N1	0.965	0.915-1.018	0.188	0.999	0.947-1.054	0.975
NX	1.188	1.096-1.287	0	1.12	1.034-1.214	0.006
SurgOthSites						
No	Reference					
Yes	0.797	0.675-0.941	0.007	0.804	0.681-0.95	0.011
Radiation						
No	Reference					
Yes	0.751	0.655-0.861	0	0.858	0.748-0.984	0.028
Chemotherapy						
No	Reference					
Yes	0.423	0.402-0.445	0	0.42	0.399-0.443	0

Uni-HR, Univariate Hazard Ratio; Uni-CI, Univariate Confidence Interval; Uni-P, Univariate P-value; Multi-HR, Multivariate Hazard Ratio; Multi-CI, Multivariate Confidence Interval; Multi-P, Multivariate P-value.

### 3.4 Nomogram-based survival prediction

**Figure 1:**
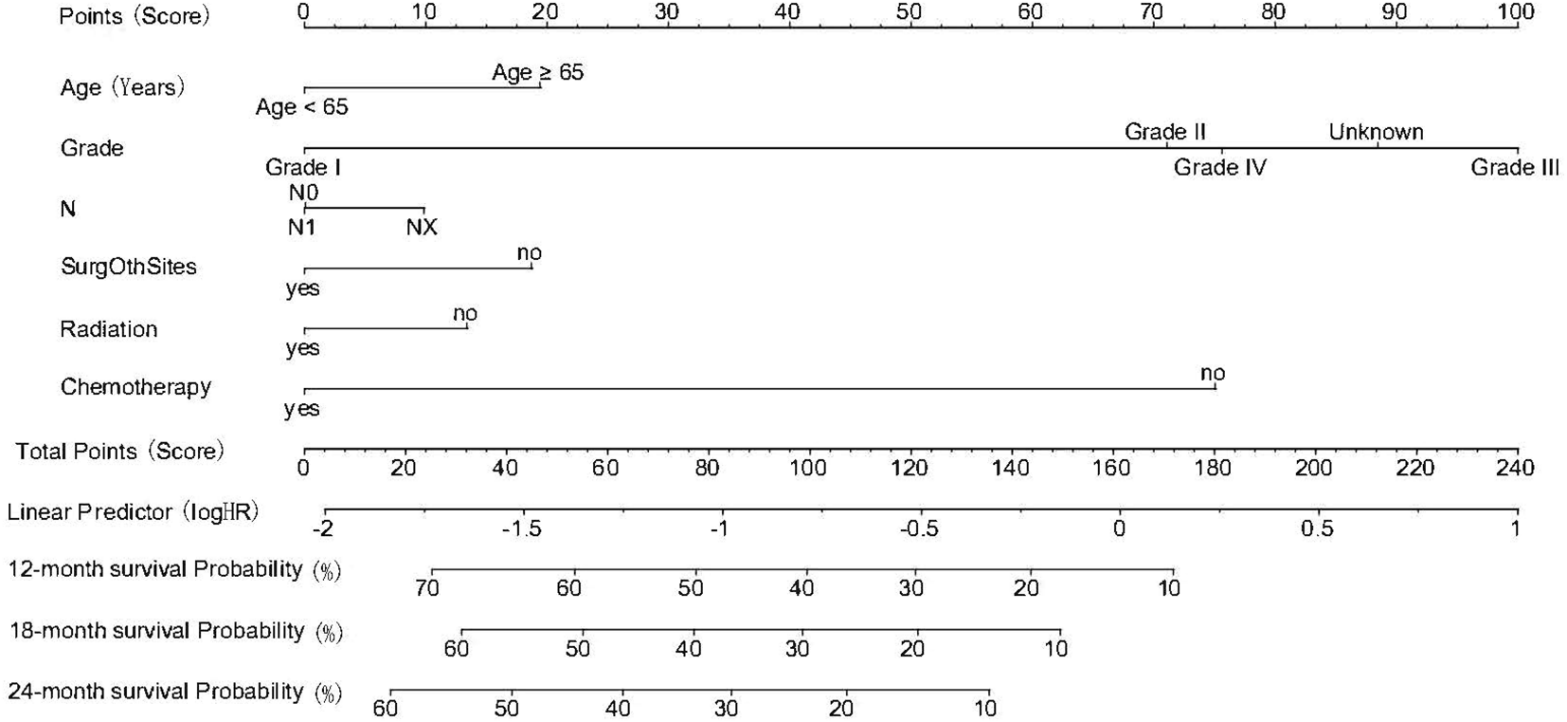
Nomogram for predicting 12-, 18-, and 24-month overall survival in patients with pancreatic cancer and synchronous liver metastases. The model integrates six independent prognostic factors: age, tumor grade, lymph node status (N), surgery at other metastatic sites, radiotherapy, and chemotherapy. Each predictor is assigned a score on the top scale; the sum of the scores corresponds to predicted survival probabilities at each time point (Age 1: <65y, Age 2≥65y; Grade 1-4: Grade I-IV; N 0-1: N0, N1; SurOthSites 0-1: SurOthSites No/Yes; Radiation 0-1: Radiation No/Yes; Chemotherapy 0-1: Chemotherapy No/Yes).

A prognostic nomogram was constructed to estimate individualized survival probabilities at 12, 18, and 24 months for patients with PDACLM based on the multivariate Cox regression results (**Figure 1**). The model incorporated six independent prognostic variables: age, tumor grade, lymph node status (N stage), surgery at other metastatic sites, radiotherapy, and chemotherapy. Each predictor was assigned a corresponding score based on its relative contribution to overall survival, as determined by the regression coefficients. Among them, tumor grade and receipt of chemotherapy had the greatest impact, reflected by their wider point ranges. By summing the individual scores for each patient, a total point value could be derived, which was then projected to a linear predictor scale and translated into predicted survival probabilities. For example, patients with higher total points—indicating the presence of unfavorable factors such as older age, poorly differentiated tumors, and lack of treatment—corresponded to lower estimated survival probabilities. In contrast, patients with favorable profiles, including lower grade tumors, younger age, and receipt of chemotherapy and local treatments, were associated with higher predicted survival at each time point. The nomogram provided a straightforward graphical interface to facilitate individualized risk estimation, allowing for a practical clinical interpretation of multiple prognostic factors in aggregate. Overall, the predicted 12-month survival probabilities ranged from 0.10 to 0.70, while 24-month survival estimates ranged from 0.10 to 0.60, reflecting substantial heterogeneity in patient outcomes within this advanced disease cohort (**Figure 1**).

### 3.5 Discriminative performance of the nomogram

**Figure 2:**
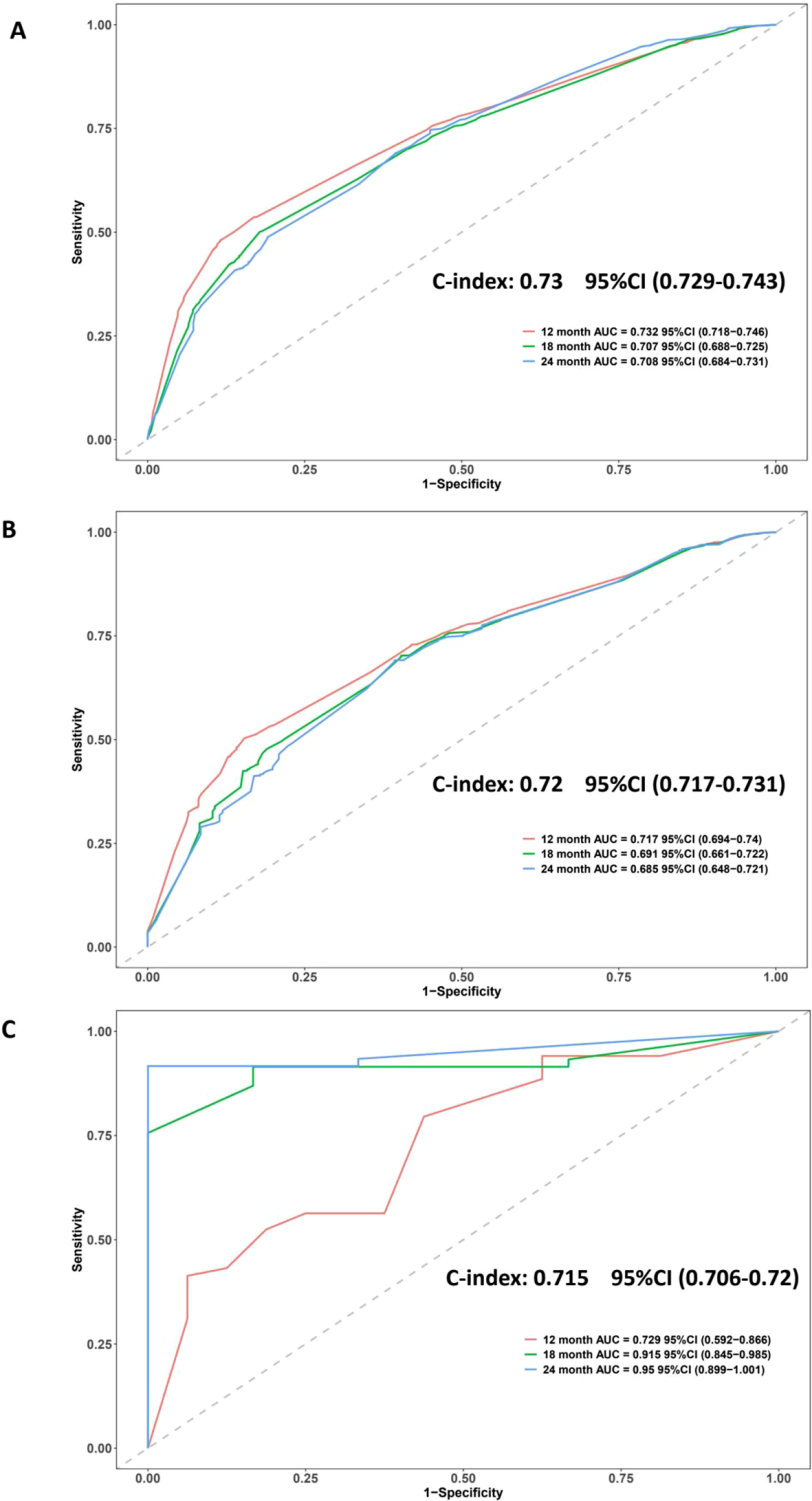
ROC curves and C-index values evaluating the discriminatory ability of the nomogram in three cohorts. **(A)** SEER training cohort: C-index = 0.73; AUCs = 0.732 (12-month), 0.707 (18-month), 0.708 (24-month). **(B)** SEER validation cohort: C-index = 0.72; AUCs = 0.717, 0.691, 0.685. **(C)** External validation cohort: C-index = 0.715; AUCs = 0.729, 0.915, 0.950. The curves indicate good model discrimination across datasets and time points.

The discriminative ability of the proposed nomogram was evaluated using time-dependent ROC curves and C-index across the training, internal validation, and external validation cohorts (**Figure 2**). In the SEER training set, the model achieved a C-index of 0.73 (95% CI: 0.729–0.743), with AUCs of 0.732, 0.707, and 0.708 for 12-, 18-, and 24-month overall survival, respectively. These results indicated a stable ability to distinguish patients with varying survival probabilities over time. Similar performance was observed in the SEER internal validation cohort, where the model yielded a C-index of 0.72 (95% CI: 0.717–0.731). The corresponding AUCs were 0.717 at 12 months, 0.691 at 18 months, and 0.685 at 24 months, demonstrating consistent model behavior within the same population source. External validation using an independent dataset confirmed the generalizability of the model, with a C-index of 0.715 (95% CI: 0.706–0.720). Notably, in the external cohort, the AUCs for 12-, 18-, and 24-month survival were 0.729, 0.915, and 0.950, respectively, suggesting increased accuracy at longer time points in that specific patient population (**Figure 2**). Moreover, despite differences in cohort size and treatment exposure, the nomogram maintained robust predictive performance across all datasets. ROC curves after stratified subsampling showed AUC values of 0.73 (training), 0.721 (internal validation), and 0.715 (external validation), indicating consistent discrimination ability. These results underscore the model’s resilience to sample distribution shifts (**Supplementary Figure 5**). Meanwhile, Time-dependent C-index curves showed consistently favorable values across all cohorts, indicating stable discriminatory performance. Time-dependent Brier scores remained low (generally <0.20) throughout follow-up, suggesting good calibration and prediction accuracy **Supplementary Figure 3**.

Time-dependent AUC analyses were performed to evaluate the dynamic discriminative ability of the nomogram over time. In the SEER training cohort, AUC values remained relatively stable between 0.80 and 0.87 throughout the follow-up period. In contrast, the external validation cohort exhibited a progressive increase in AUC over time. This phenomenon is likely due to a smaller sample size and fewer late events in the external cohort, leading to potential overestimation of long-term discrimination accuracy (**Supplementary Figure 6**). And ten-fold cross-validation showed stable C-index values across all folds (median: 0.771, interquartile range: 0.765–0.779), indicating good internal consistency and low risk of overfitting (**Supplementary Figure 7**).

### 3.6 Calibration of the nomogram

**Figure 3:**
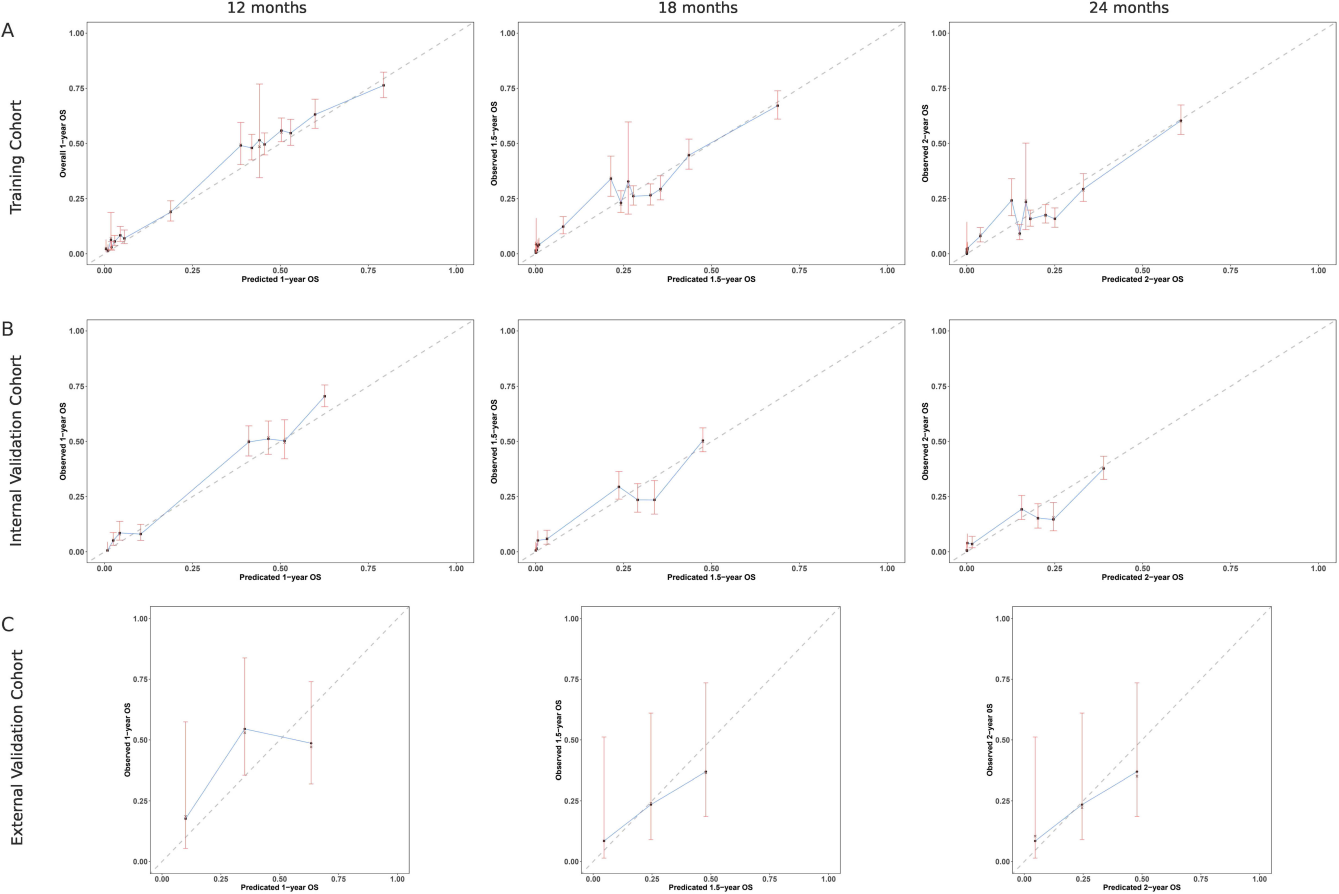
Calibration curves comparing predicted and observed overall survival (OS) at multiple time points. **(A)** SEER training cohort: calibration at 12, 18, and 24 months shows good agreement with the ideal line. **(B)** SEER internal validation cohort: similar consistency observed at the same time points. **(C)** External validation cohort: calibration assessed at 12 months indicates strong alignment with observed OS. The dashed line represents perfect prediction (45°), and vertical bars indicate 95% confidence intervals.

The calibration performance of the nomogram was evaluated by comparing the predicted and observed OS probabilities at 12, 18, and 24 months across different datasets (**Figure 3**). In the SEER training cohort (**Figure 3A**), the calibration curves showed a close agreement between predicted and actual survival, with most data points aligning near the 45-degree reference line. This indicated accurate survival estimation across a wide probability range. The internal validation cohort (**Figure 3B**) demonstrated similar patterns, with calibration curves showing good consistency across all time points, although wider confidence intervals were observed in the upper prediction range, suggesting slight variability at higher survival probabilities. In the external validation cohort (**Figure 3C**), calibration was performed for 12-month survival and revealed a strong correlation between predicted and observed values, despite a smaller sample size (**Figure 3**).

### 3.7 Clinical benefit based on decision curve analysis

**Figure 4:**
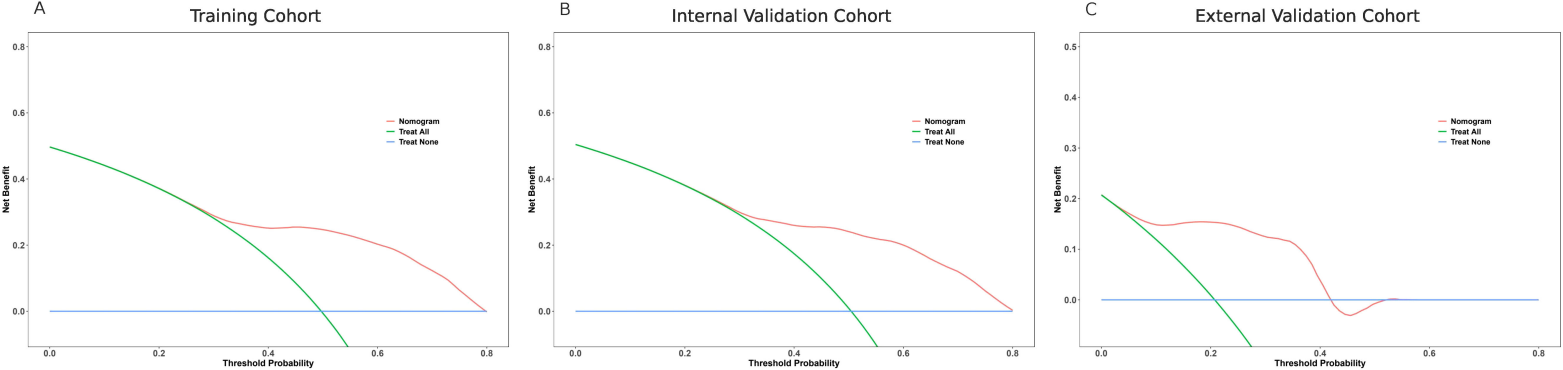
Decision curve analysis (DCA) of the nomogram in three cohorts. **(A)** SEER training cohort; **(B)** SEER internal validation cohort; **(C)** External validation cohort. The red line represents the net benefit of using the nomogram across different threshold probabilities, compared with treating all patients (green line) or none (blue line). The nomogram shows superior net benefit over a wide range of thresholds in all cohorts.

The clinical applicability of the nomogram was assessed using DCA across the training, internal validation, and external validation cohorts (**Figure 4**). In the SEER training cohort (**Figure 4A**), the nomogram provided a higher net benefit than either the “treat all” or “treat none” strategies when the threshold probability ranged from approximately 0.1 to 0.65. This pattern was similarly observed in the internal validation cohort (**Figure 4B**), where the model consistently outperformed default strategies across a wide threshold range, indicating reliable clinical value in guiding decision-making. In the external validation cohort (**Figure 4C**), although the overall net benefit observed in the external validation cohort was relatively modest, likely reflecting the smaller sample size and limited number of long-term events, the nomogram still showed a potential positive net benefit within the threshold probability (**Figure 4**). Specifically, DCA showed that Model A consistently yielded greater net benefit than the TN staging model (Model B) in both the training and internal validation cohorts, especially within the clinically relevant threshold range of 0.2 to 0.6. A similar trend was observed in the external validation cohort, despite its limited sample size (n = 82), suggesting potential generalizability while highlighting the need for further validation in larger external datasets (**Supplementary Figure 8**).

### 3.8 Survival analysis in clinical subgroups

**Figure 5:**
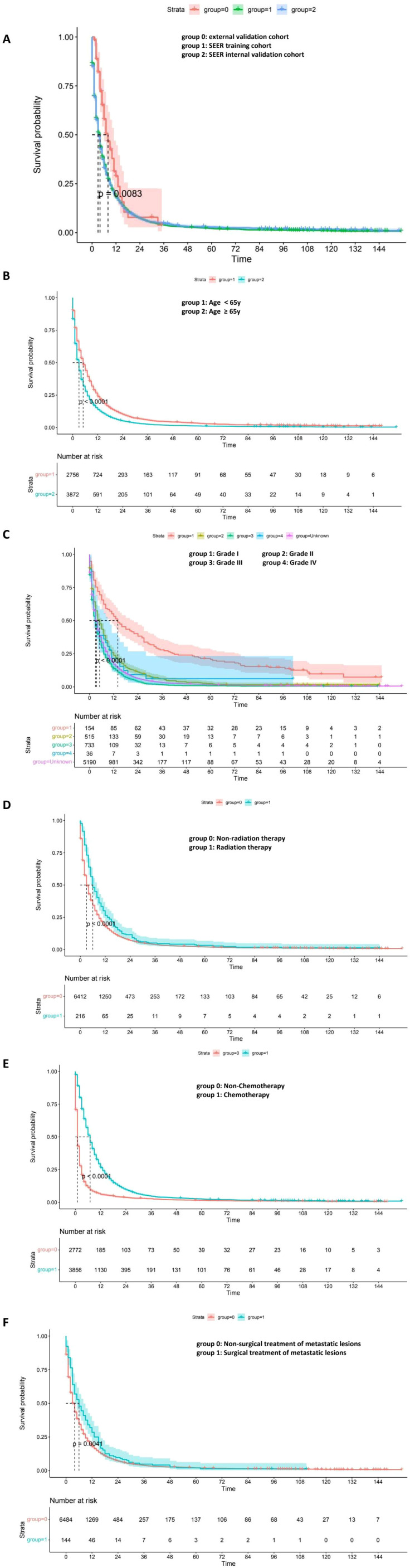
Kaplan–Meier survival curves depicting survival outcomes across clinical subgroups. **(A)** Survival comparison among cohorts: external validation (group 0), SEER training (group 1), and SEER internal validation (group 2). **(B)** Age <65 years (group 1) vs. ≥65 years (group 2). **(C)** Tumor grade: Grade I (group 1), Grade II (group 2), Grade III (group 3), Grade IV (group 4), unknown grade (group: unknown). **(D)** Non-radiation (group 0) vs. radiation therapy (group 1). **(E)** Non-chemotherapy (group 0) vs. chemotherapy (group 1). **(F)** Nonsurgical (group 0) vs. surgical treatment of metastatic lesions (group 1). Log-rank *P* values are shown in each panel.

To explore the prognostic relevance of individual clinical variables, subgroup survival analyses were conducted using Kaplan–Meier curves (**Figure 5**). In the cohort comparison (**Figure 5A**), statistically significant survival differences were observed among the external validation, SEER training, and SEER internal validation groups (*P* = 0.0083), indicating some inter-cohort variability. Age-based stratification (**Figure 5B**) showed that patients aged ≥65 years had significantly worse overall survival compared to younger patients (*P*<0.0001). Tumor differentiation also exhibited a strong association with prognosis (**Figure 5C**): survival declined progressively from Grade I to Grade IV, with the poorest outcomes seen in poorly differentiated and undifferentiated tumors (*P*<0.0001). **Figure 5D** demonstrated that patients receiving radiotherapy had improved survival compared to those who did not (*P*<0.0001), but its prognostic effect was not exclusive to the external group. Furthermore, no significant interaction between cohort and radiotherapy was observed in the Cox model (P = 0.11), indicating no differential treatment effect across cohorts. Furthermore, despite a markedly higher radiotherapy rate in the external cohort (21.95% vs. 3.26%), PSM analyses showed that survival differences persisted after balancing key covariates, suggesting that treatment imbalance alone did not fully account for the observed outcomes (**Supplementary Figure 4**) and the model demonstrated relatively stable predictive performance across datasets, suggesting potential applicability in broader clinical contexts, though further prospective validation is warranted. In addition, **Figure 5E** indicated a similarly pronounced survival advantage in patients who underwent chemotherapy (*P*<0.0001). Additionally, surgical treatment of metastatic lesions (**Figure 5F**) was associated with modestly prolonged survival relative to nonsurgical management (*P* = 0.0041). Across all subgroup comparisons, the observed survival differences were statistically significant and consistent with the predictors included in the nomogram, further reinforcing their clinical and prognostic relevance (**Figure 5**).

### 3.9 Clinical utility of risk stratification and surgical status

To explore clinical translation, we stratified patients in each cohort into low- and high-risk groups based on the nomogram, and further analyzed outcomes by metastatic site resection status. Kaplan–Meier curves consistently showed that low-risk patients who underwent surgery had the most favorable survival, whereas high-risk patients without surgery had the poorest prognosis. Notably, among high-risk patients, survival differences between those with and without surgery were minimal across all cohorts, suggesting limited benefit from metastasectomy in this subgroup. These findings highlight the potential role of risk stratification in guiding individualized treatment planning (**Supplementary Figure 9**).

Stratified Kaplan–Meier analysis demonstrated that the survival benefit associated with metastasectomy was primarily observed in the low-risk group, whereas patients in the high-risk group derived limited benefit. This suggests that favorable outcomes following surgery were not uniformly distributed and may reflect biologic selection rather than surgical efficacy alone (**Supplementary Figure 10**).

## 4 Discussion

In this study, we identified key prognostic factors and constructed a validated nomogram to estimate survival outcomes in patients with PDACLM who did not undergo curative surgery. Using a large, population-based SEER cohort alongside an external Chinese validation set, we found that older age (≥65 years), higher tumor grade, and unknown lymph node status were independently associated with poorer overall survival, while chemotherapy, radiotherapy, and metastasis-directed surgery conferred significant survival benefits. The developed nomogram, integrating these clinical and treatment variables, demonstrated strong discriminative power (C-index: 0.73) and calibration across both internal and external cohorts. Importantly, the model captured the heterogeneity of survival outcomes in this historically under-characterized patient population, enabling individualized risk stratification and supporting its potential utility in guiding personalized treatment decisions in advanced PDAC.

Due to the anatomical connection via the portal vein, PDAC cells that invade blood vessels often metastasize to the liver (27). Despite progress in medical technology, the prognosis for PDAC has not significantly improved over the past decade (28). Although its prevalence remains stable at around 3%, the mortality rate has increased from 6% in 2012 to 8% in 2022 (29). For patients with PDACLM who respond favorably to chemotherapy and are physically fit for surgery, subsequent resection may be beneficial (30, 31). However, most patients with extensive hepatic involvement are not surgical candidates, which remains a key factor behind the dismal prognosis in this population (32). Historically, research on metastatic PDAC has focused on those undergoing conversion therapy or achieving resection after neoadjuvant treatment. For instance, Barenboim et al. (33) highlighted advanced PDAC after NAT was the only preoperative radiological factor that predicted adverse survival in patients undergoing curative surgery after FOLFIRINOX, while Jaoude et al. (34) reported stereotactic body radiation therapy could also be considered in advanced PADC patients in cases where the tumor does not invade the duodenum or other neighboring structures. Nevertheless, these studies largely neglect patients who, despite receiving intensive systemic therapy, ultimately fail to meet surgical criteria due to disease progression or declining clinical condition. Our study specifically addresses this overlooked group, capturing real-world complexity and avoiding the resectability bias that skews previous prognostic models (35, 36).

Importantly, our results reaffirm that chemotherapy and radiotherapy can provide meaningful survival benefits even among non-surgical candidates. The observed advantages associated with the resection of metastatic lesions suggest that multidisciplinary strategies may help selected patients, though this may reflect selection bias, such as better functional status, fewer metastases, or more indolent tumor biology (37–39). In contrast to models dependent on molecular or radiomic data (20, 40) and external cohort’s higher radiotherapy rate raised concerns about potential treatment-related bias. However, multiple sensitivity analyses suggested that this imbalance, while notable, did not invalidate model performance. This approach, in lieu of traditional undersampling, helped mitigate bias and supports the model’s generalizability across heterogeneous populations and suggest that observed survival differences may reflect broader regional practice patterns rather than model limitations. In the meantime, the inclusion of time-dependent performance metrics further strengthens our model’s prognostic validity. Despite the relatively small external validation sample, both time-dependent C-index and Brier curves remained within acceptable ranges, supporting generalizability and mitigating concerns of overfitting. Thus, our nomogram is based on routinely available clinical variables to make it suitable for broader clinical application and DCA showed consistently higher net benefit of Model A versus TNM staging across cohorts, suggesting potential clinical utility and generalizability,similarly, in the external cohort, despite limited size, a similar trend supported model robustness; however, cautious interpretation is warranted, and further validation in larger, diverse populations remains necessary. By stratifying survival in this high-risk, unresectable cohort, our model helps bridge a crucial gap in PDAC prognostic assessment and supports individualized decision-making that goes beyond a simple surgical/non-surgical dichotomy (41, 42). Moreover, Kaplan–Meier analysis revealed a significant survival difference between cohorts (P = 0.0083), which diminished after multivariable adjustment (HR = 1.061, P = 0.1885). Subsequent risk stratification showed that patients who received radiotherapy or metastasectomy were predominantly from the low-risk group, suggesting possible treatment selection bias(**Supplementary Figure 10**). These results indicate that the observed survival gap is more likely attributable to regional differences in clinical practice and baseline characteristics rather than limitations of the model itself. Similar findings have been reported in other SEER-based models with external validation, where inter-cohort differences attenuated after adjusting for covariates (43, 44). Notably, most NX patients had poorly differentiated tumors (grade III–IV), which may indicate more aggressive disease or advanced stage where nodal dissection was either not feasible or deemed clinically unnecessary. These findings align with prior studies (45, 46) that have similarly reported adverse outcomes associated with NX status, highlighting that NX may represent a surrogate marker for disease severity rather than simple staging omission. Furthermore, similar findings have been noted in SEER-based studies of other cancers, where NX was linked to worse outcomes (46). Interestingly, N stage was significant in the original dataset but not after multiple imputation (47). This may result from imputation smoothing, which reduces the impact of partially missing variables (48, 49).

Current guidelines from the American Society of Clinical Oncology (ASCO) and the National Comprehensive Cancer Network (NCCN) emphasize systemic therapy as the primary treatment for metastatic PDAC, while curative-intent surgery is reserved for a highly selective group of patients with favorable tumor biology and good response to induction regimens (11, 41). Interestingly, although some earlier studies identified radiotherapy as a risk factor for bone metastases in PDAC, our results indicate that it may be beneficial for patients with liver metastases (42). In particular, radiotherapy using radioactive particle implantation has demonstrated therapeutic potential in PDACLM patients (50, 51), supporting its role not only as a palliative measure but also as a possible conversion strategy for otherwise unresectable cases with liver involvement. Beyond local control, recent advances in molecular oncology have enabled precision-based systemic strategies. Patients harboring *BRCA* or *PALB2* mutations benefit from platinum-based chemotherapy or PARP inhibitors (52), while those with *MSI-H/dMMR* tumors may respond favorably to immune checkpoint inhibitors such as pembrolizumab, with emerging evidence supporting combination with radiotherapy (53). Additionally, rare *NTRK* fusion–positive cases are amenable to TRK inhibitor therapy (54). Expanding on this, experimental combinations—such as immunotherapy plus radiotherapy and MEK inhibition—have shown promise in biologically selected subgroups (55). However, these clinical frameworks often lack detailed recommendations for the substantial population that remains ineligible for surgery despite intensive multimodal therapy (42).

Our nomogram specifically targets this intermediate-risk group, helping to fill the gap between curative and purely palliative approaches. By integrating accessible clinical indicators—such as tumor grade, nodal status, and prior treatment exposure—the model offers a more refined approach to evaluating therapeutic potential, aligned with emerging principles of precision oncology (40). Rather than relying solely on anatomical staging or binary surgical criteria, it enables clinicians to tailor treatment intensity to individual prognostic profiles. Furthermore, to evaluate the added predictive value of our nomogram, we compared it against the conventional AJCC TNM staging system using C-index analysis. Our model demonstrated substantially higher discriminative ability in both the training (C-index: 0.77 vs. 0.50) and validation (0.76 vs. 0.51) cohorts. These findings underscore the importance of incorporating tumor biology and treatment exposure into individualized risk prediction, particularly in patients with advanced unresectable disease where traditional staging may have limited applicability. In addition, nomogram-based stratification yielded distinct survival curves across risk groups. Kaplan–Meier analysis showed that low-risk patients benefited most from metastasectomy, whereas high-risk patients gained limited survival advantage. These findings highlight the nomogram’s clinical utility in guiding surgical decision-making.

Despite the strengths of our study, several limitations must be acknowledged. As a retrospective analysis, inherent selection bias is unavoidable, and unmeasured confounders—such as performance status, comorbidities, or specific chemotherapy regimens—may have influenced survival outcomes (56). This study is subject to limitations inherent to its retrospective design. Although propensity score matching was applied to reduce selection bias, unmeasured confounding remains a concern. To mitigate this as much as possible, we incorporated proxy variables (age, tumor grade, and treatment exposure) and conducted stratified survival analyses combining risk group with surgical status. Only low-risk patients demonstrated significant benefit from metastasectomy, suggesting that the observed survival differences were not solely attributable to treatment selection. Additionally, sensitivity analyses including multiple imputation, 10-fold cross-validation, and external validation confirmed the model’s stability. Nonetheless, prospective validation in datasets with more granular clinical information is warranted. In addition, the SEER database, while extensive, lacks detailed data on emerging molecular biomarkers (KRAS, BRCA mutations), radiomic characteristics, and treatment-related toxicities, all of which could enhance risk stratification (20). The external validation cohort, although valuable for testing model generalizability, was relatively small and drawn from a single institution, which may limit its applicability across broader populations or healthcare settings. Moreover, the unusually high 24-month AUC observed in this cohort may reflect overestimation due to limited follow-up and a low number of long-term events, potentially inflating discrimination metrics at later time points. This should be interpreted with caution. Similarly, in the external validation cohort, the DCA curve suggested net benefit only within a narrow probability range (5–41%), likely due to the limited sample size and follow-up. While such findings constrain potential clinical conclusions, they still align with decision thresholds meaningful for unresectable PDACLM (57). To improve the robustness of prognostic modeling, future research should incorporate molecular and imaging biomarkers alongside clinical variables to build integrated, multi-modal prediction tools. Large-scale prospective validation across diverse, multi-center cohorts is also essential to confirm the nomogram’s effectiveness in real-world practice.

## 5 Conclusion

In conclusion, our study presents a clinically applicable nomogram for prognostic assessment in patients with PDACLM who ultimately remain ineligible for curative resection—a group often underrepresented in existing prognostic frameworks. By incorporating easily accessible clinical and treatment-related variables, this model offers a practical approach to individualized survival estimation and may assist clinicians in stratifying therapeutic priorities. While the findings are encouraging, particularly in highlighting the potential survival benefits of multimodal therapy even among non-surgical candidates, they should be interpreted with caution due to the retrospective nature of the data and the relatively limited size of the external validation cohort. Further prospective validation across larger and more diverse populations is needed to confirm the robustness and clinical utility of this tool before widespread implementation.

## Data Availability

The original contributions presented in the study are included in the article/**Supplementary Material**. Further inquiries can be directed to the corresponding author.
