# Supplementary figures and images for: Development and validation of a prognostic nomogram for unresectable pancreatic ductal adenocarcinoma with synchronous liver metastases: a study based on the SEER database and an external cohort
Source: Front Oncol. 2025 Aug 27;15:1636715. doi: 10.3389/fonc.2025.1636715 (PMC12420269; doi:10.3389/fonc.2025.1636715)

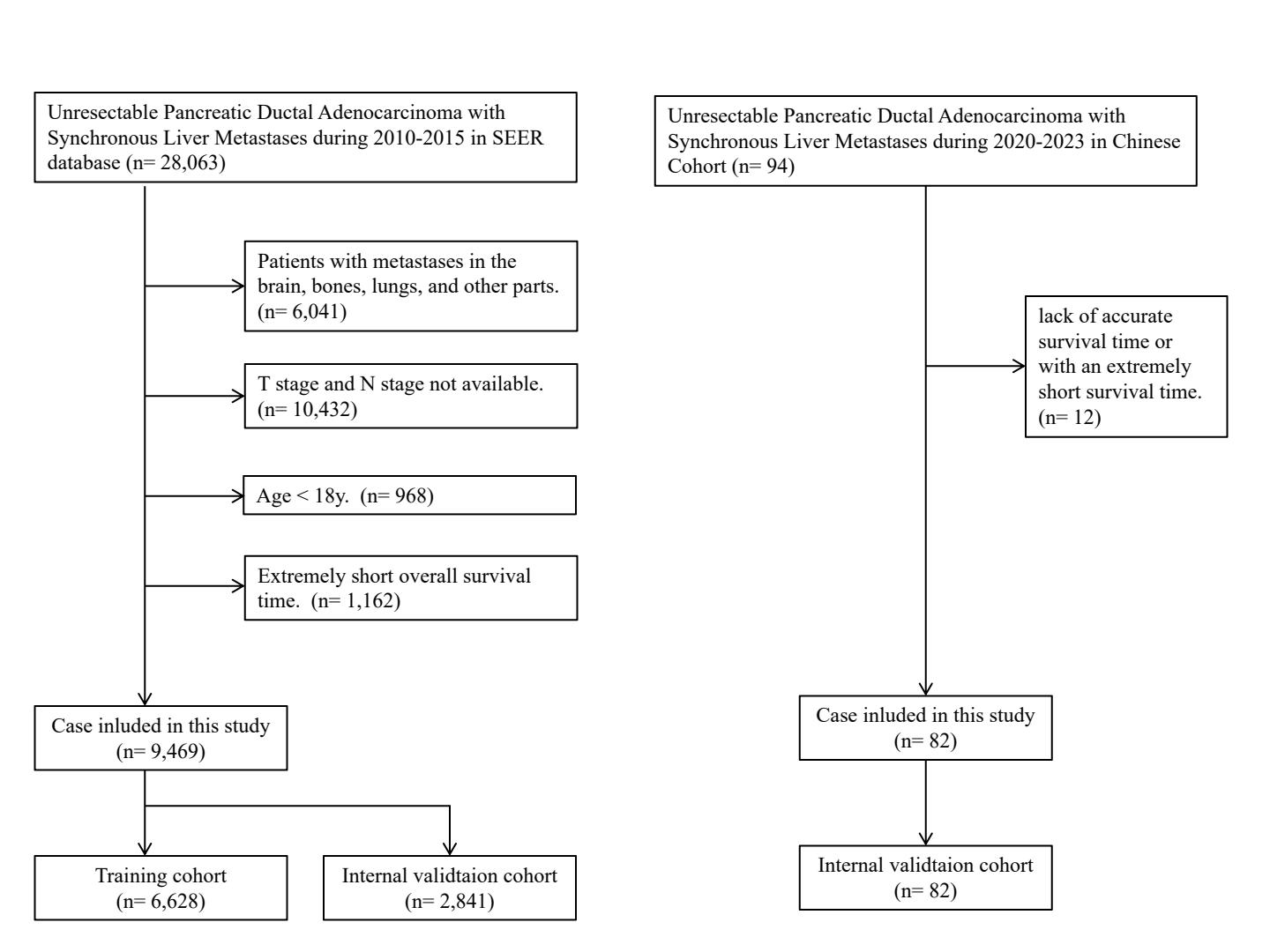

Supplement: Supplementary file 1 [file Image1.jpeg]

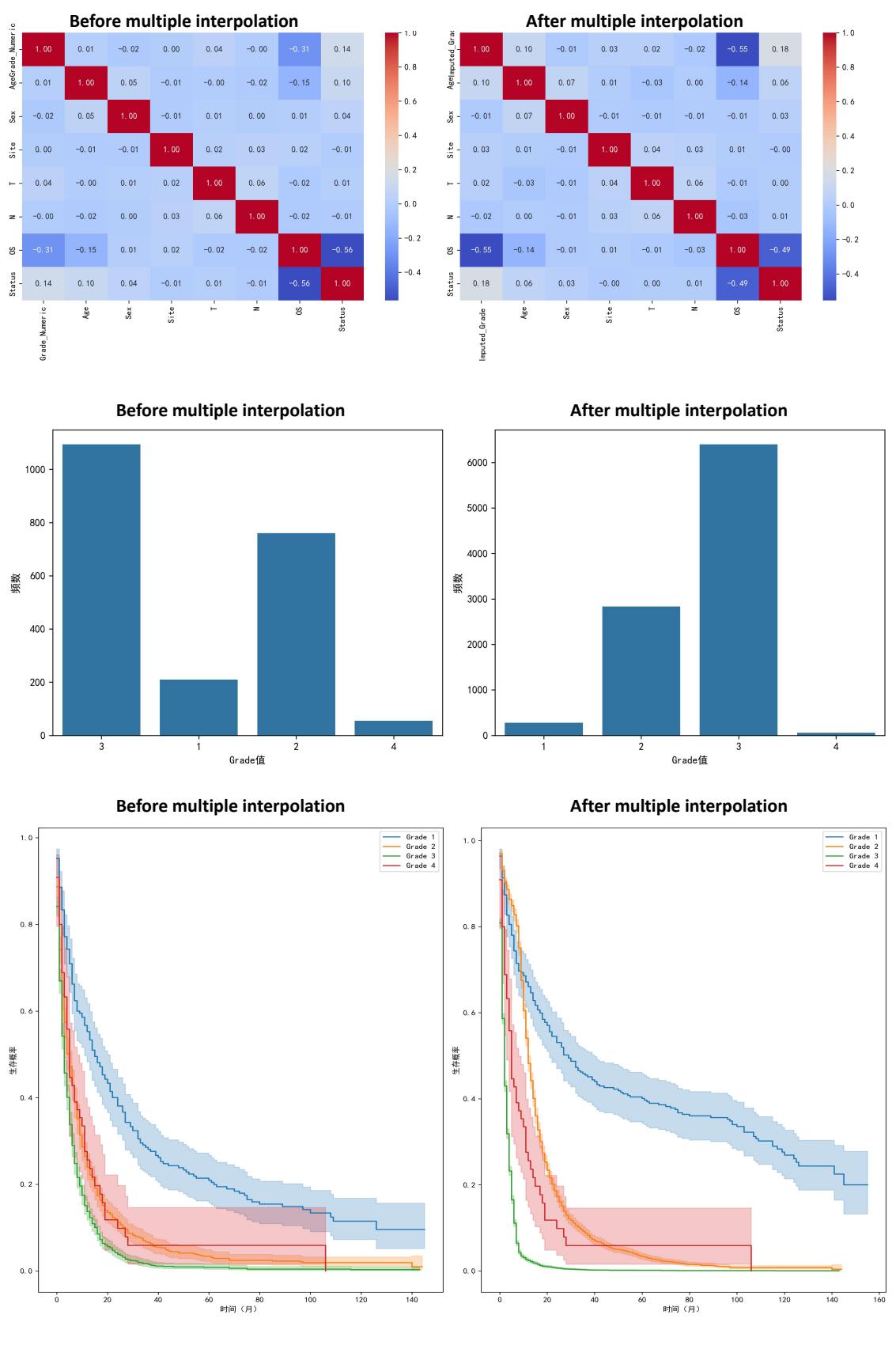

Supplement: Supplementary file 2 [file Image2.jpeg]

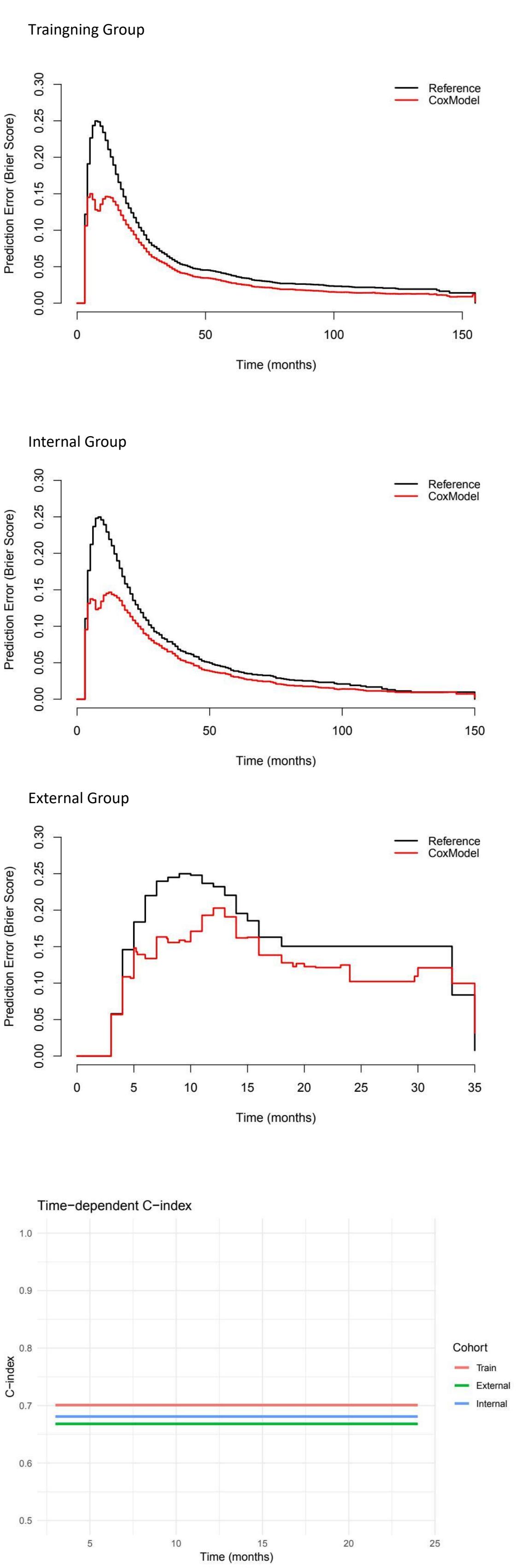

Supplement: Supplementary file 3 [file Image3.jpeg]

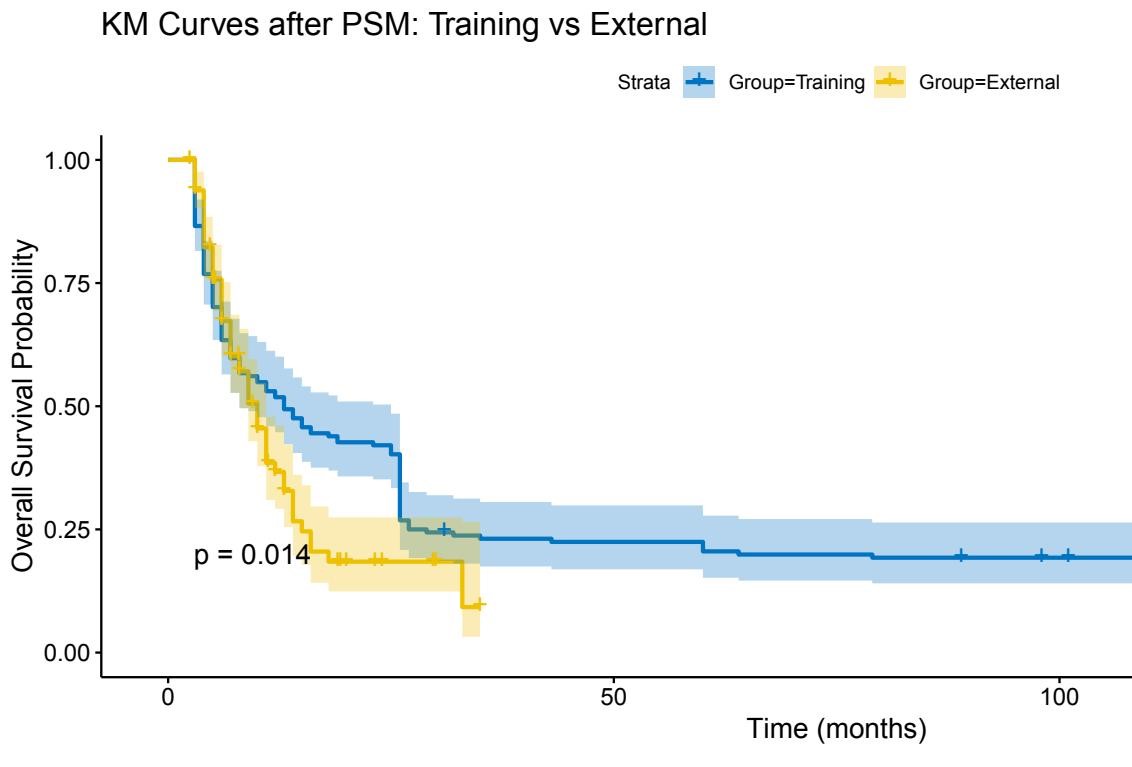

Supplement: Supplementary file 4 [file Image4.jpeg]

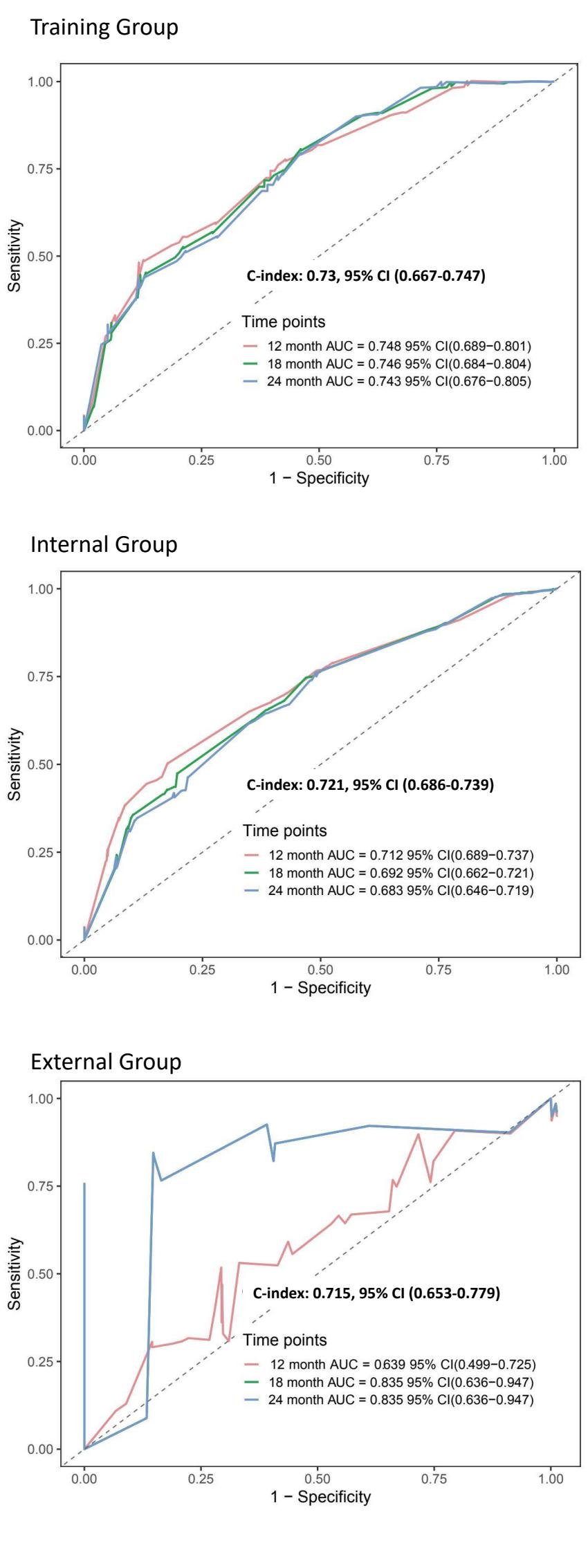

Supplement: Supplementary file 5 [file Image5.jpeg]

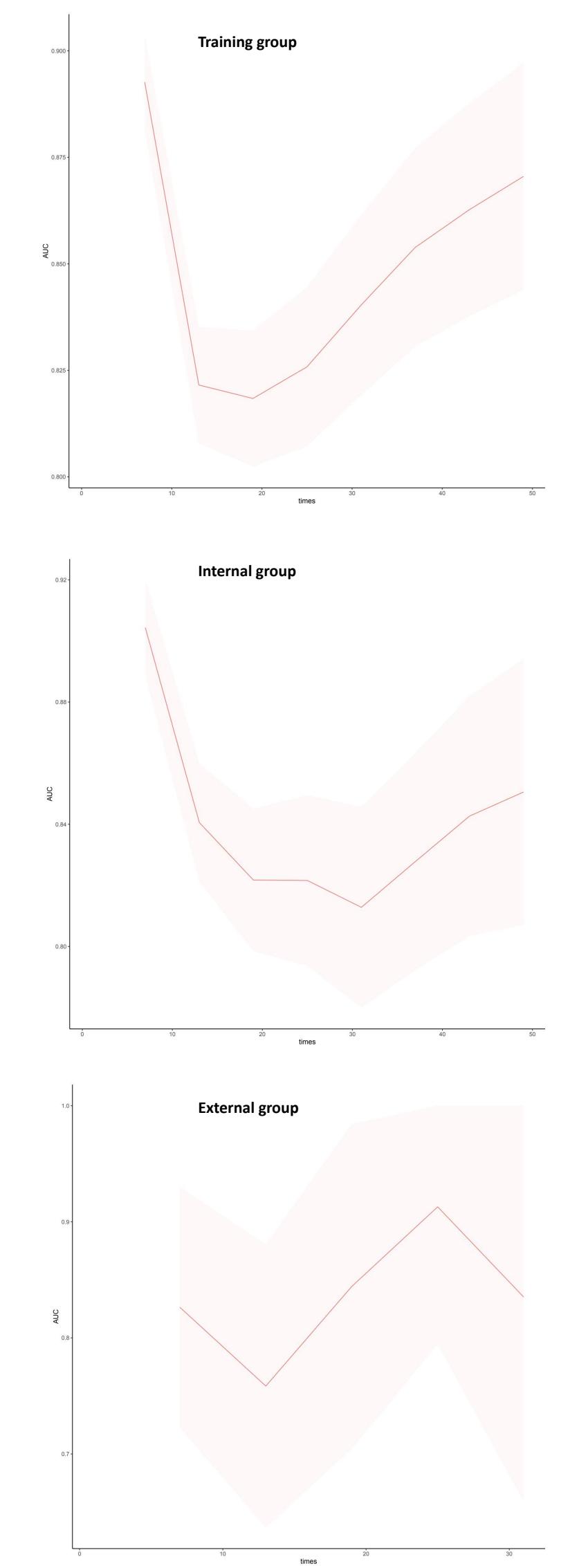

Supplement: Supplementary file 6 [file Image6.jpeg]

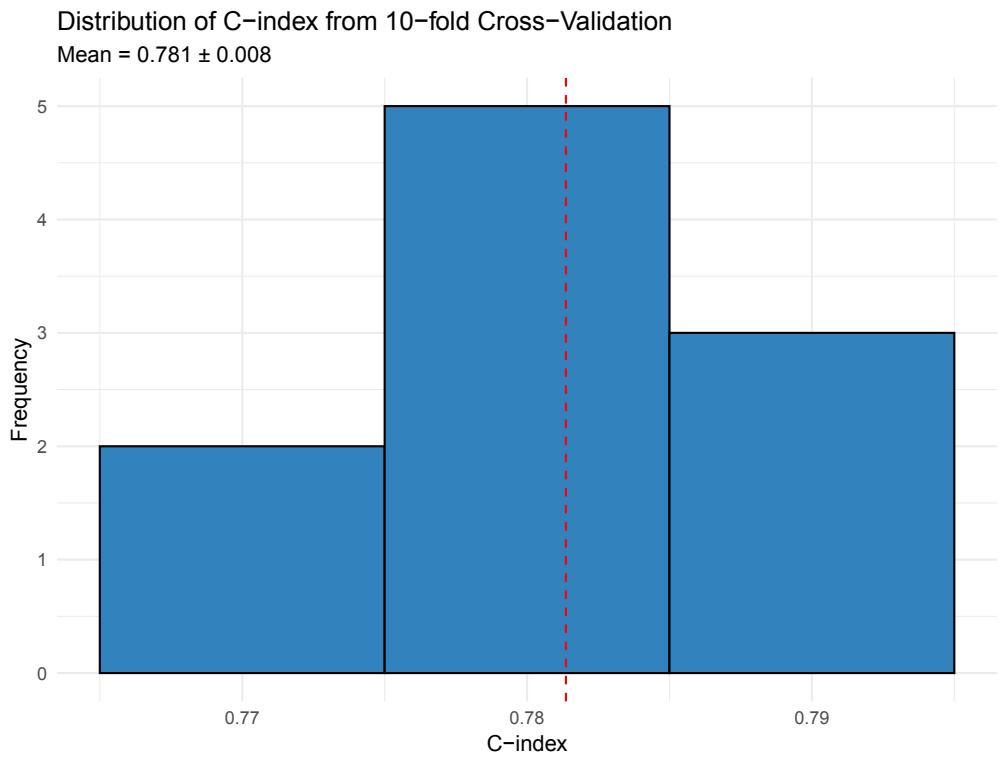

Supplement: Supplementary file 7 [file Image7.jpeg]

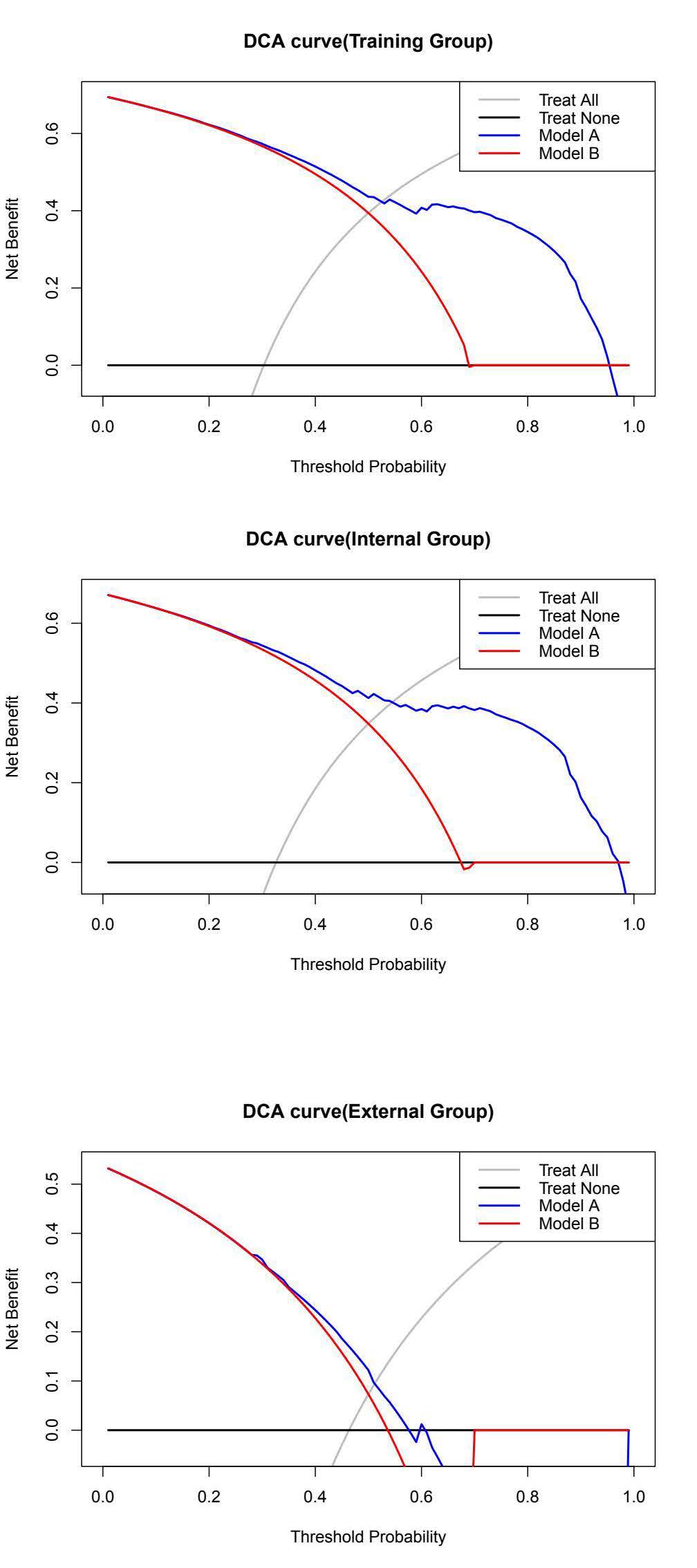

Supplement: Supplementary file 8 [file Image8.jpeg]

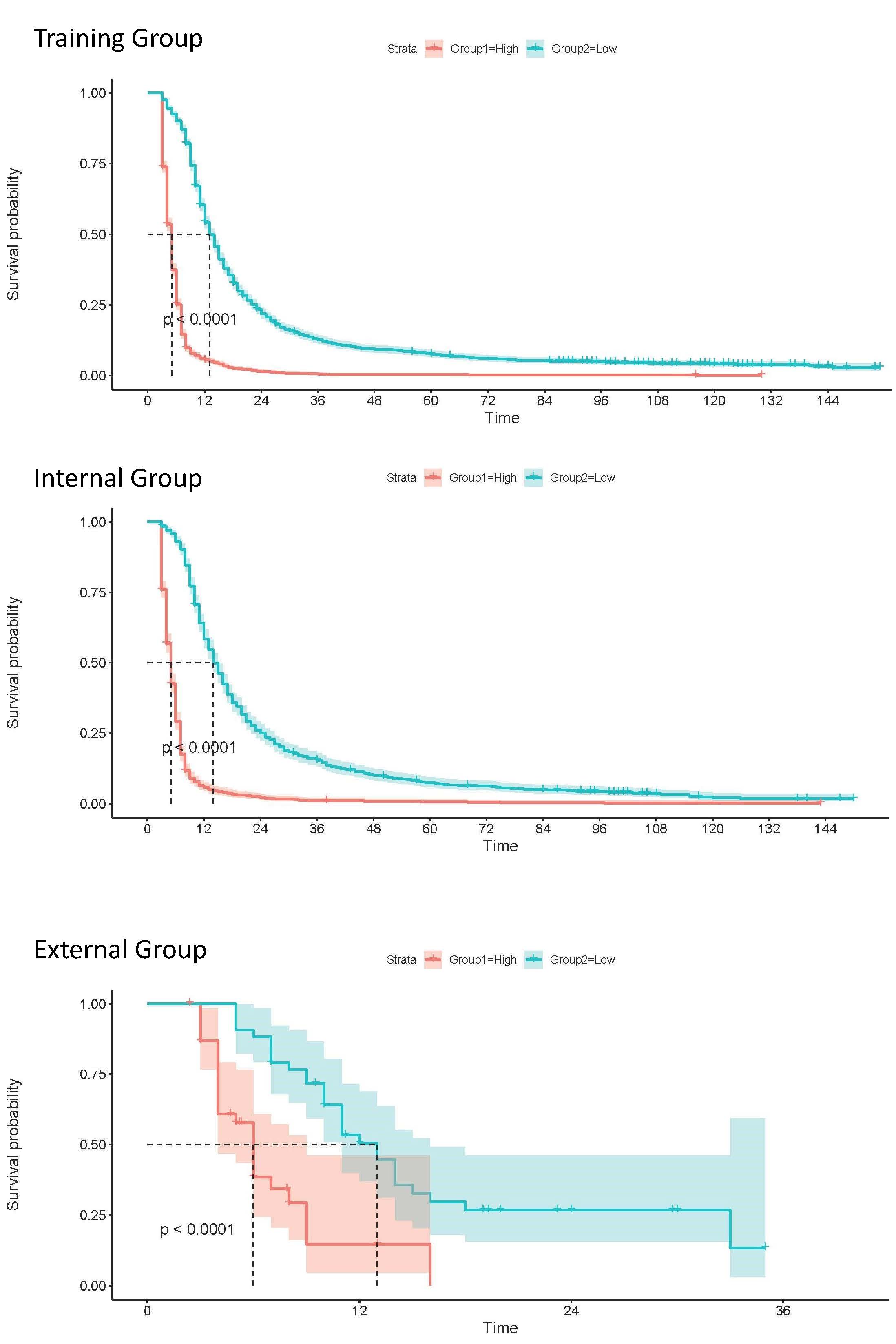

Supplement: Supplementary file 9 [file Image9.jpeg]

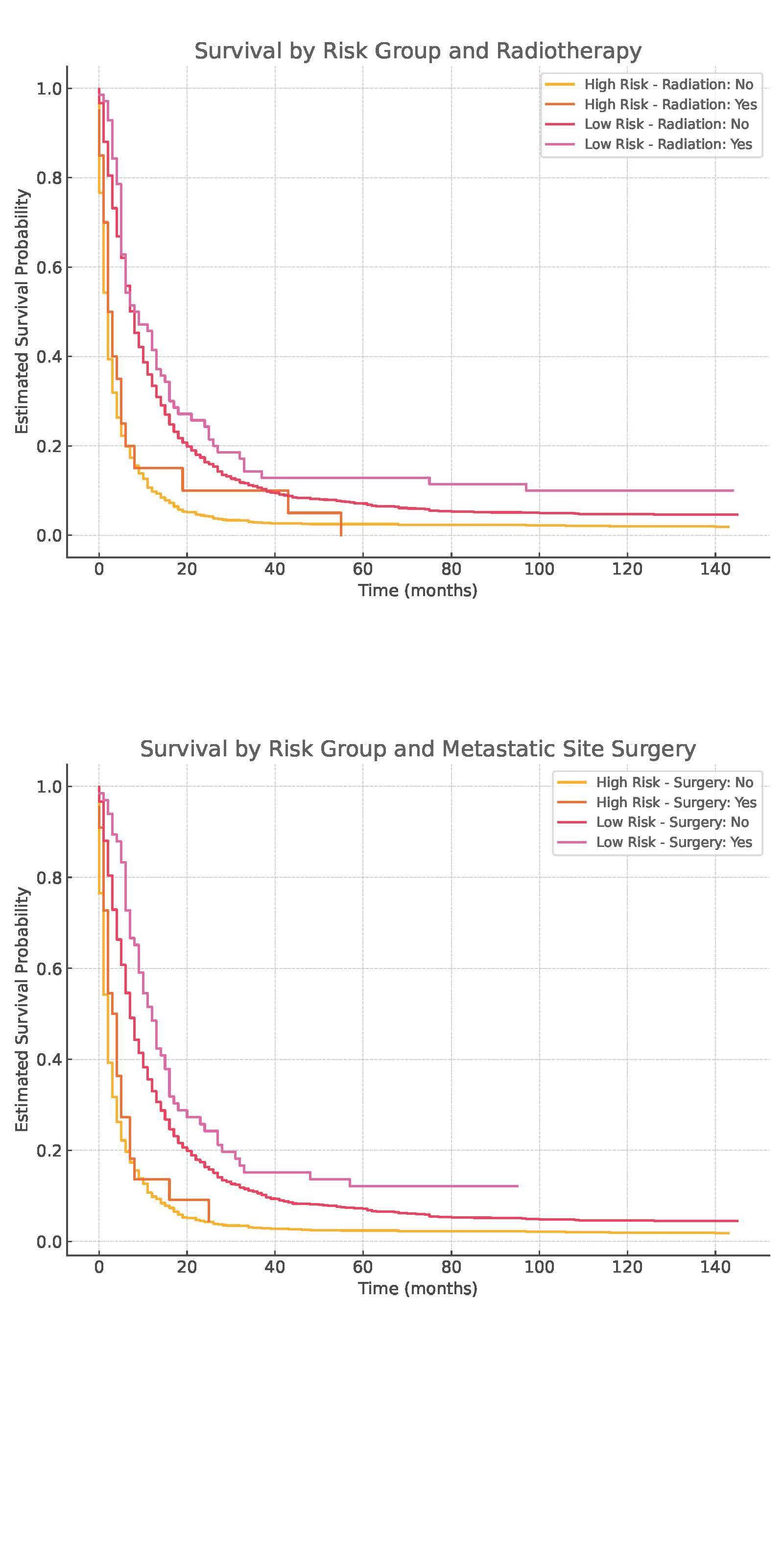

Supplement: Supplementary file 10 [file Image10.jpeg]
